# Feasibility of incorporating genomic knowledge into electronic medical records for pharmacogenomic clinical decision support

**DOI:** 10.1186/1471-2105-11-S9-S10

**Published:** 2010-10-28

**Authors:** Casey Lynnette Overby, Peter Tarczy-Hornoch, James I Hoath, Ira J Kalet, David L Veenstra

**Affiliations:** 1 Department of Medical Education & Biomedical Informatics, University of Washington, Seattle, WA, USA; 2 Department of Pediatrics, University of Washington, Seattle, WA, USA; 3 Department of Computer Science & Engineering, University of Washington, Seattle, WA, USA; 4 Medical Center Information Services, University of Washington, Seattle, WA, USA; 5 Department of Radiation Oncology, University of Washington, Seattle, WA, USA; 6 Biological Structure, University of Washington, Seattle, WA, USA; 7 Department of Pharmacy, University of Washington, Seattle, WA, USA

## Abstract

In pursuing personalized medicine, pharmacogenomic (PGx) knowledge may help guide prescribing drugs based on a person’s genotype. Here we evaluate the feasibility of incorporating PGx knowledge, combined with clinical data, to support clinical decision-making by: 1) analyzing clinically relevant knowledge contained in PGx knowledge resources; 2) evaluating the feasibility of a rule-based framework to support formal representation of clinically relevant knowledge contained in PGx knowledge resources; and, 3) evaluating the ability of an electronic medical record/electronic health record (EMR/EHR) to provide computable forms of clinical data needed for PGx clinical decision support. Findings suggest that the PharmGKB is a good source for PGx knowledge to supplement information contained in FDA approved drug labels. Furthermore, we found that with supporting knowledge (e.g. IF age <18 THEN patient is a child), sufficient clinical data exists in University of Washington’s EMR systems to support 50% of PGx knowledge contained in drug labels that could be expressed as rules.

## Introduction

With the promise of personalized medicine, there is a push for genomics-based diagnostics to be incorporated into clinical practice [1]. Genomic medicine is medical practice that incorporates genomics-based diagnostics and has the potential to facilitate personalized medicine. In the pursuit of personalized medical practice, pharmacogenomic (PGx) information that may be incorporated into guidelines for prescribing drugs based on a persons’ genotype, is of particular interest in this research. If clinical data and PGx knowledge can be taken into account to improve initial drug dosage for individuals, improvements in patients’ outcomes may be achieved in a cost-effective manner. Here we evaluate the feasibility of incorporating PGx knowledge, combined with clinical data to support clinical decision-making.

The objectives for our study were to: 1) Perform an analysis of clinically relevant knowledge contained in PGx knowledge resources; 2) Evaluate the feasibility of a rule-based framework to support formal representation of clinically relevant knowledge contained in PGx knowledge resources; and, 3) Evaluate the ability of an electronic medical record/electronic health record (EMR/EHR) to provide computable forms of the clinical data needed for PGx clinical decision support.

## Related work

PGx has already entered into clinical practice [2]. Here, we give an overview of some existing standards for presenting external knowledge in EMRs; and, describe some resources that provide evidence necessary for making PGx-related decisions.

### Just-in-time clinical decision support

There are several instances in which the just-in-time metaphor has been used in the context of medical decision-making. One example is the Infobutton Manager [3], an information resource that is accessed through a clinical information system, anticipates clinician’s questions, and provides links to pertinent electronic resources. Another example is the MINDscape electronic health record (EHR) system [4], a Web-based integrated interface that provides access to both patient specific information and knowledge resources that contain information such as drug reference information and clinical guidelines. Both of these approaches provide point-of-care access to knowledge, and both focus on methods for automatically selecting and retrieving appropriate knowledge resources. The MINDscape system, developed at the University of Washington (UW), is of particular relevance to this research. We will use this system as an example EHR framework against which we will evaluate the feasibility of incorporating PGx knowledge for clinical decision support. In this study we analyze PGx knowledge resources including PharmGKB [5], FDA approved drug labels [6], and relevant biomedical literature. Objectives 2 and 3 focus on characterizing PGx knowledge contained in drug labels in particular.

### Existing pharmacogenomic knowledge resources

The pharmacgenetics and pharmacogenomics knowledge base (PharmGKB, http://www.pharmgkb.org) is an online, publicly available, resource that contains multiple forms of curated PGx information to generate knowledge of relationships among genes, drugs, and diseases. Of interest to this work are the curated publications containing evidence to support relationships; information concerning the effects of genetic variation on relationships; and, drug-centered pathways connecting genes involved in pharmacokinetics (PK) and pharmacodynamics (PD) with highly curated knowledge and primary data[7].

There are three studies to date that have examined the availability of PGx information in drug labels [8-10]. In 2006, researchers reported a lack of specific PGx-based recommendations for prescribing and dosing of drugs [8]. Of the top 200 prescribed drugs, they found that 71.3% had published PGx information in the literature, but only three had package inserts with PGx information sufficient to guide individualized dosing. In a 2008 study, authors reported that although there remains a gap between published information on PGx and PGx information found in labels, drug approvals have recently included more PGx information [10]. In the analysis, they demonstrate that one fourth of all prescriptions are for drugs that contain PGx information in their labeling. There are now a small number of products in the United States for which the FDA have associated mandatory or recommended genetic tests [6]. Within the product labels, the listed drugs have PGx information under several sections including: “Clinical Pharmacology,” “Indications and Usage,” “Warnings or Precautions,” and/or “Dosage and Administration.”

## Methods

Toward our goal to analyze the feasibility of representing existing PGx knowledge for clinical decision support in computable form, we had three objectives. Specific methods for each objective are described below.

### Availability of clinically relevant knowledge

For our first objective, we perform an analysis of clinically relevant information contained in PGx knowledge resources. Specifically, we review the drug labels of 28 primary drugs from the FDA “Table of valid genomic biomarkers in the context of approved drug labels” (FDA biomarker-drug pairs) [6]; the supporting references listed for each FDA biomarker-drug pair; references identified in PharmGKB that contain evidence of the biomarker-drug relationship; and, the types of electronically available knowledge produced by the FDA and contained within PharmGKB.

For each drug, we record the associated publications containing evidence of biomarker-drug relationships on the FDA website and within PharmGKB and determine the degree of overlap of evidence coverage in these resources. In addition, for each drug we catalogue the curated knowledge contained in PharmGKB. Forms of curated knowledge defined by PharmGKB include: *categories of evidence*, *pathway evidence*, *variant evidence*, *genotypedata*, *phenotype data*, and *clinical PGx section*[5].

The PGx literature is curated using five *categories of evidence* and standardized vocabularies of genes, drugs and diseases. These categories include: clinical outcome (CO); pharmacodynamics (PD); pharmacokinetics (PK); molecular and cellular functional assays (FA); and, genotype (GN). In the PGx literature, CO, PD, PK and FA are forms of phenotypic evidence. *Pathway evidence* includes knowledge of biochemical pathways associated with the use of a particular medication. *Variant evidence* includes knowledge of genetic variants associated with individual response to therapy. *Genotype* and *phenotypedata* designate the existence of these types of data. C*linical PGx section* designates drugs for which all related knowledge has been compiled within PharmGKB.

We classify electronically available knowledge as *encoded*, *tagged,* or *computable*. Encoded and tagged information may be described as computer accessible, and computable knowledge as computer-readable. An example of *tagged information* would be a wiki page that contains a table of contents. The information on the wiki page is free text, but tagged for particular sections identified in the table of contents. Resources containing *encoded information* utilize controlled vocabularies in order to add structure to information and facilitate more complex computer access. For example, PubMed contains encoded information about publication authors, titles, journals, etc. A user is then able to specify encoded data as search terms and execute complex queries across all publications. *Computable knowledge* is knowledge described in a language for communication with the computer. If knowledge is computable, then it can be described as an algorithm or rule, and implemented as such in a computer program or application. We discuss some of the steps taken to translate PGx knowledge into computable form in the next section.

### Feasibility of rule-based representation

For our second objective, we evaluated the feasibility of representing PGx knowledge in a computable form, suitable to code within an EHR framework. In our previous objective, we catalogued the different types of electronically available knowledge produced by the FDA and contained in PharmGKB. Steps required to translate knowledge encoded in the PharmGKB into a computable form would differ from steps to translate FDA drug label content, due to differences in the type of information represented and the form in which it is captured (i.e. free text, tagged, encoded).

For example, encoded knowledge about enzyme substrates contained in PharmGKB (e.g. S-Warfarin is a substrate of CYP2C9) could be utilized in a clinical decision support rule or in an algorithm. With supplemental knowledge about CYP2C9 variants (e.g. CYP2C9*2 and CYP2C9*3 variants are associated with impaired substrate metabolism); and drug specific knowledge (e.g. When taking Warfarin, decrease in S-warfarin clearance is associated with increased bleeding risk), we can define decision support rules such as “IF patient is taking Warfarin AND patient has CYP2C9*2 or CYP2C9*3 variants, THEN there will be a decrease in S-warfarin clearance AND there is an increased bleeding risk.” However, here we focus on FDA drug labels because they are created to provide clinical guidance, and are therefore more likely to contain all of the knowledge necessary to define a clinical decision support rule.

For the 28 drug labels of the FDA biomarker-drug pairs, we identified passages containing clinically relevant knowledge that may support PGx clinical decisions; and, wherever feasible, translate passages into an if-then rule representation. PGx knowledge contained in drug labels are primarily in free-text form, therefore these steps are performed manually.

In addition, we cluster PGx knowledge into general categories and determine the type of user interface (UI) presentation that would be appropriate. An example category may be knowledge that provides support for determining “who should be screened for a genetic variant prior to administering a particular treatment.” UI presentation types characterize how actionable a *then* statement is, of an if-then rule. Types include: *information only*; *recommendation;* and, *warning alert*. A statement is classified as *information only* if no direct action is specified within the statement, or actions are specified using language with a low degree of certainty (i.e. might, may, could). Conversely, a statement is classified as a *recommendation* if a clear action is specified using language with a medium to high degree of certainty (i.e. should, will, are, is, must, was, do); and as a *warning* if potential consequences are specified (language may be of any degree of certainty). In cases where a statement falls into multiple categories, a choice is made according to the following prioritization: warning, or information, or recommendation. That is, if a statement is identified as being both a recommendation and a warning, it is classified as a warning. Similarly, if a statement is identified as both information only and a recommendation, it is classified as a recommendation. Each designation refers to the type of presentation that is appropriate given how actionable the if-then rule.

### Availability of computable patient data

For our final objective, we evaluated the ability of an EMR to provide in computable form (not free text) clinical data needed for PGx clinical decision support. Particularly, we looked at data available in the UW MINDscape EHR system, including laboratory systems (pathology and microbiology), and performed the following steps for each of the if-then rules we identify in Objective 2: 1) determine the types of clinical data needed in combination with PGx knowledge to provide clinical decision support; 2) determine whether or not different types of data are already captured as discrete data in the EMR; and, 3) for clinical data that are not currently captured, estimate the feasibility of capturing these data by expert opinion.

As an example, clinical data needed for the Warfarin rule, described in the previous section, would be the inclusion (or considering the inclusion) of Warfarin on a patients’ medication list; and, CYP2C9 variant status. In some cases, our ability to utilize clinical data might depend on the existence of supporting knowledge. For example, with the statement “the patient is a poor metabolizer of CYP2C9,” supporting knowledge must define the CYP2C9 genotype that would classify a patient as “a poor metabolizer of CYP2C9,” (e.g. IF patient has genotype CYP2C9*2/*3 THEN the patient is a poor metabolizer).

Lastly, given the availability of clinical data and the if-then rules that represent clinically relevant PGx knowledge that we identify, we evaluate the ability for these rules to be executed in the UW clinical care environment. For each if-then rule we determined whether all clinical data needed to execute the rule exists as discrete data. Cases where all data needs are satisfied can be directly implemented in the UW EMR environment. For cases where all data needs are not met, we evaluated the feasibility of including the required clinical data. Clinical data types considered most feasible to include within the UW EHR framework by expert opinion are: disease status definitions; data fields in pathology laboratory systems; data fields in microbiology laboratory systems; and pathology or microbiology laboratory values that exist in free text and can be parsed without use of full natural language processing (NLP) methods. “Feasible expansion” represents the inclusion of these clinical data types within the UW EMR system. When appropriate, initially unsatisfied clinical data needs can be categorized as satisfied with “feasible expansion” of the UW EMR system.

## Results

Results of the evaluation across three objectives are described below.

### Availability of clinically relevant knowledge

Related to our first objective, we found that there was little overlap between references containing evidence of biomarker-drug relationships: 1) identified by the FDA; and, 2) identified within PharmGKB. There were 185 articles containing evidence listed on the FDA website and 268 articles with evidence of biomarker-drug relationships of interest contained in the PharmGKB. Only 28 (6.4%) of the total set of articles containing evidence were found in both the PharmGKB database and on the FDA website. Of the 28 contained in both resources, 11 (39%) were not designated as containing evidence of the particular drug-biomarker relationship of interest in PharmGKB.

In addition, of the 251 publications in PharmGKB that do not overlap with those listed by the FDA, the representation of categories of evidence for biomarker-drug relations are as follows: CO – 49(19%); PD – 80(32%); PK – 141(56%); FA – 27(11%); and, GN – 171(68%). These designations of “evidence categories” may be useful for expanding upon the repository of biomedical literature containing knowledge to support PGx clinical decisions.

In our evaluation of electronically available knowledge for drug labels and relevant literature, we found that all but one drug label (Codeine sulfate) could be located in the DailyMed online database[11]. DailyMed contains electronically available knowledge in which drug label sections are tagged, but data are still in free text. In addition to DailyMed drug sections, PharmGKB could be a useful source of PGx knowledge relevant to guide the use of each drug. Table 1 displays types of encoded knowledge available for each drug.

**Table 1:** Types of encoded knowledge in PharmGKB.

	**pathway evidence**	**variant evidence**	**genotype data**	**phenotype data**	**clinical PGx section**
**Abacavir**		x			x
**Atomoxetine**					
**Atorvastatin**	x	x			x
**Azathioprine**		x	x	x	x
**Busulfan**					
**Capecitabine**		x			
**Carbamazepine**		x			x
**Celecoxib**	x	x			
**Cetuximab**	x				x
**Clopidogrel**	x	x			x
**Codeine sulfate**	x	x			
**Dasatinib**					x
**Erlotinib**	x	x			
**Fluoxetine HCL**	x	x		x	
**Imatinib mesylate**	x	x			x
**Irinotecan**	x	x	x	x	x
**Isoniazid**	x				
**Lenalidomide**					
**Maraviroc**					
**Nilotinib**					
**Panitumumab**					x
**Prasugrel**					
**Primaquine**					
**Pyrazinamide**	x				
**Rasburicase**					x
**Rifampin**	x	x	x	x	
**Trastuzumab**		x			x
**Valproic acid**	x				x
**Voriconazole**					
**Warfarin**	x	x	x	x	x

Types of PharmGKB encoded knowledge for medications that also contain PGx knowledge in their drug labels.

Overall, we found that PharmGKB is a good supplement to FDA approved drug label contents. PharmGKB contains encoded knowledge from a large number of publications that may provide additional evidence to support PGx relationships contained in drug labels. However, this knowledge is not currently in a computable format. In objective 2, we explore our ability to represent PGx knowledge in a format suitable to code within an EHR framework.

### Feasibility of rule-based representation

Related to our second objective, from the 28 drug labels reviewed, we were able to identify 79 passages containing knowledge to support PGx clinical decisions. We were able to translate all but 2 passages into one or more decision support rules. In total, we defined 106 if-then rules to support PGx clinical decisions based on currently recommended FDA PGx testing.

**Figure 1:**
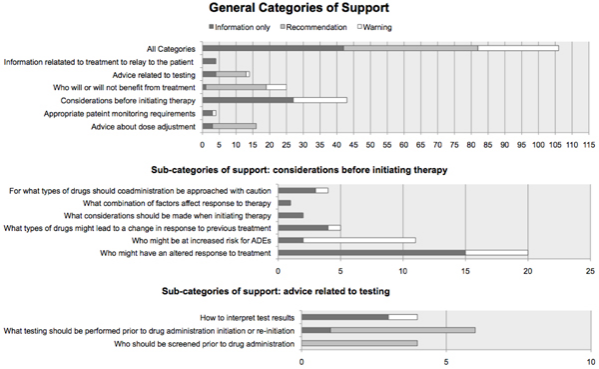
**General and sub- categories of clinical decision support**. Counts for if-then rules clustered into general and sub- categories; and representation of UI classifications (information only, recommendation, and warning alerts) within each category.

In further evaluation we found that, given the status of a patient with respect to a biomarker, 50% of all approximate decision support rules help clinicians make decisions about dose adjustments; help clinicians determine appropriate patient monitoring requirements; and/or illuminate certain considerations (e.g. risk of adverse drug events, or potential altered response) before initiating therapy. In addition, 24% of the rules provide advice on who will or will not benefit from treatment; 13% provide advice related to testing (e.g. “how should test results be interpreted”); 9% provide advice on coadministration of medications (e.g. “for what types of drugs should coadministration be approached with caution”); and, 4% provide advice on what information related to treatment should be relayed to the patient (See Figure 1 for details on categories of clinical decision support provided by rules).

Furthermore, we determined the type of UI presentation that would be appropriate for each rule. We found that 39% should be presented as *information only*; 39% should be presented as a *recommendation*; and, 22% should be presented as a *warning* (See Table 2 for examples, and Figure 1 for details on representation of presentation types within various categories of support).

**Table 2:** Example if-then rules.

Approximate decision support rule	Inpatient/ Outpatient DB	Laboratory DB	Supportive Knowledge	Information/ Recommendation/ Warning
IF the patient has ASM AND the patient has a tumor with a D816V c-Kit mutation THEN recommended dose of Gleevec is 400 mg/day	Medication – Imatinib mesylate (Gleevec®) Disease status – ASM, DFSP, or GIST	Tumor/Pathogen genotype – gastrointestinal stromal tumor c-Kit expression	N/A	Recommendation

IF the patient has a CYP2C9 variant AND the variant causes poor metabolism THEN the dose of Celecoxib should be reduced by 50%	Medication – Celecoxib	CYP2C9 variant status	CYP2C9 variants causing poor metabolism	Recommendation

IF the patient has a CYP2C19 variant AND the variant causes poor metabolism THEN the dose adjustment for Clopidogrel is unknown for the patient	Medication – Clopidogrel (Plavix®)	CYP2C19 variant status	CYP2C19 variants causing poor metabolism	Information

IF the patient has hepatic impairment OR the patient is taking a medication that is a strong CYP2D6 inhibitor OR the patient is a CYP2D6 poor metabolizer THEN the dose of Atomoxetine should be adjusted	Disease status – hepatic impairment Medication – Atomoxetine Medication list	CYP2D6 variant status	Medications that are CYP2D6 inhibitors CYP2D6 variants causing poor metabolism	Information

IF the patient will be taking Fluoxetine AND the patient is taking other drugs that are metabolized by CYP2D6, THEN coadministration should be approached with caution	Medication – Fluoxetine HCL (Prozac®) Medication list	N/A	Medications metabolized by CYP2D6	Warning

Example if-then rules for PGx clinical decision support.

### Availability of computable patient data

**Figure 2:**
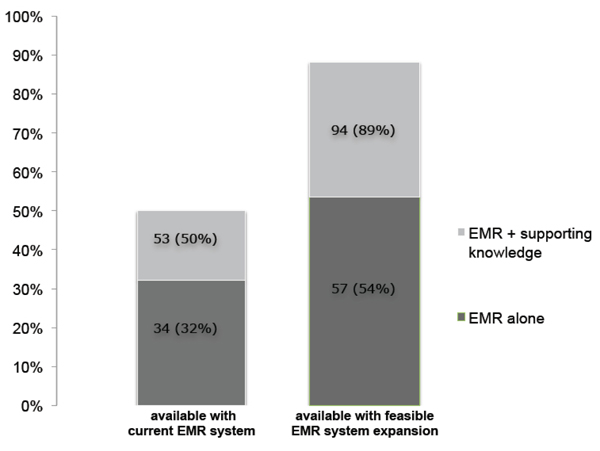
**Clinical data access for rule execution**. Both “EMR alone” and “EMR + supported knowledge” represent accessible clinical data within an EMR. Supporting knowledge defines classifications such as “IF age < 18 THEN patient is a child.”

Related to our third objective, given the current availability of clinical data, we found that 32% of our 106 if-then rules could be expressed without additional supporting knowledge or information contained in clinical notes (See Figure 2). The addition of supporting knowledge would raise the percentage of rules with sufficient clinical data access to 50%. We also determined, by expert opinion, the feasibility of expanding the current UW EMR system to incorporate data fields that allow for the execution of PGx clinical decision support rules. We considered “feasible expansion” to be the addition of disease status definitions (11%); the addition of data entry fields in pathology laboratory systems (<1%); the addition of data fields in microbiology laboratory systems (20%); and, the ability to parse (not by full natural language processing) pathology or microbiology laboratory values that exist in free text (7%). Percentages designate instances within the full set of if-then rules for which lack of access to these definitions/fields would inhibit our ability to execute rules. We found that with feasible expansion to the current EMR system, sufficient clinical data access for our if-then rules would increase to 89%.

## Limitations

Definitions and assignments of if-then rules into general categories (as described in the methods for **Feasibility of Rule-based Representation** section), were performed by a single author. We evaluate the consistency of assignments through informal review of classifications by all authors. While assignments are generally agreed upon, in order to establish interrater reliability we would need to assess agreement among multiple persons’ assigning if-then rules to the various categories.

## Discussion

Overall, we found that our research findings will be useful for designing a model to incorporate PGx knowledge into EMR systems for clinical decision support. In our first objective, we evaluated PharmGKB and FDA drug labels as potential sources for PGx knowledge in the context of clinical practice, and found that PharmGKB might be a good supplement to FDA drug labels as a source for evidence to support PGx clinical decisions.

However, PGx knowledge encoded in PharmGKB alone might not be considered enough evidence to guide clinical decisions. An evidence-based process incorporating rigorous evidence review strategies [12] would be ideal for making clinical recommendations. Depending on the *strength* of evidence, a choice must be made between providing information only; presenting a warning; or, recommending an action. An approach to clarifying the strength of evidence might be to incorporate the opinions of clinical and drug experts. For example, recent work with the Drug-Interaction Knowledge Base (DIKB) incorporates an evidential approach to knowledge representation [13]. They combine an evidence taxonomy containing 36 evidence types (e.g. retrospective studies, clinical trials, metabolic inhibition identification, etc.) with a set of inclusion criteria. When given a specific set of evidence, the inclusion criteria enables drug experts to specify their confidence level in a drug mechanism assertion. Such an approach might also be applied more generally to presenting PGx knowledge within an EMR, although integration with existing evidence-based processes used by healthcare systems that utilize the EMR would be an essential component of the implementation of this system.

In support of PharmGKB as a supplemental source for clinical evidence, previous work shows that PharmGKB can facilitate the assessment of the clinical validity and utility of PGx tests through the identification of relevant peer-reviewed manuscripts [14]. Manuscripts included in PharmGKB are compared against those used in evaluations performed by professional organizations like EGAPP (Evaluation of Genomic Applications in Practice and Prevention) and ACCE (Analytic validity, Clinical validity, Clinical utility, and Ethical, legal, and social implications). Manuscripts included as primary data sources in EGAPP and ACCE reviews were considered to be prototypical. Similarly, in addition to drug labels, professional organizations such as these are good sources for clinical guidance for using PGx knowledge. The organizations ascertain whether or not there is sufficient evidence to support the use of PGx tests in clinical practice by expert opinion. A recent example is a statement from the American Society of Clinical Oncology (ASCO) that recommends KRAS screening prior to initiating anti-epidermal growth factor receptor monoclonal antibody therapy (e.g. Cetuximab) [15].

In our second and third objectives, we focused on drug labels that contain PGx knowledge. We presented a scheme for translating PGx knowledge into if-then rules, a format suitable to code within an EHR framework; we evaluated the current state of PGx knowledge (i.e. how mature/actionable); and determined whether EMRs have the right data to support the execution of rules for PGx clinical decision support. We found that 50% of these rules could be executed within the UW EMR environment given the current availability of clinical data and with the addition of supporting knowledge; however, the maturity of the knowledge differs. Warning and recommendation alert messages are appropriate for 22% and 39% (respectively) of all rules derived from drug labels containing PGx knowledge. Even then, the level of certainty expressed in the language used in these statements varies.

Another interesting finding from our final objectives was that only 13% of the rules we define represent knowledge about when to perform a genetic test or how to interpret test results. Therefore, in addition to PharmGKB, resources such as the GeneReviews Knowledge Base (genetests.org) [16] might also be considered as a potential resource to supplement PGx knowledge contained in drug labels.

While results from these objectives may differ between institutions, we believe our methods are generalizable and can be used to evaluate the availability of clinical data to support PGx clinical decision support within any EHR framework. Also, it has been shown that different representations of PGx test results (e.g. gene single nucleotide polymorphisms, gene alleles) with automated interpretation (e.g. ‘homozygous normal’, ‘heterozygous affected’) can be used effectively within the EMR without impacting reaction times in responding to alert messages [17]. Therefore, we believe our methods are applicable independent of PGx data representation.

Our findings form the basis for future work wherein we will implement if-then rules electronically. As an important caveat, although recent studies have shown computerized alerts to be non-disruptive to the workflow of clinicians [18], alerts that raise frequent or insignificant alarms will likely be ignored [19,20]. Therefore, choice of triggers (clinical events that cause rules to be invoked) and rules to execute in the context of an EMR environment should be approached with care.

## List of abbreviations used

ACCE: Analytic validity, Clinical validity, Clinical utility, and Ethical, legal, and social implications; ASCO: American Society of Clinical Oncology; CO: Clinical outcome; DIKB: Drug Interaction Knowledge Base; EGAPP: Evaluation of Genomic Applications in Practice and Prevention; EHR: Electronic Health Record; EMR: Electronic Medical Record; FA: Functional assays; FDA: US Food and Drug Administration; GN: Genotype; NLP: Natural Language Processing; PGx: Pharmacogenomics; PK: Pharmacokinetics; PD: Pharmacodynamics; UI: User interface; UW: University of Washington.

## Competing interests

The authors declare that they have no competing interests.

## Authors' contributions

PTH and CLO came up with the concept and design of the approach taken. JIH determined the availability of clinical data in the UW EMR system and the feasibility of expanding the current UW EMR system to incorporate additional data fields. IJK provided feedback on methods for defining and evaluating rules derived from drug labels. PTH and DLV reviewed and provided feedback on results from the analysis of clinically relevant items contained in drug labels. CLO conducted the evaluation and prepared the manuscript. All authors participated in draft revisions. All authors contributed to, read and approved the final manuscript.
